# Lower limb motor function and hip muscle weakness in stroke survivors and their relationship with pelvic tilt, weight-bearing asymmetry, and gait speed: A cross-sectional study

**Published:** 2020-01-05

**Authors:** Vishakha Darak, Suruliraj Karthikbabu

**Affiliations:** Department of Physiotherapy, Manipal College of Health Professions, Manipal Academy of Higher Education, Manipal Hospital, Bangalore, India

**Keywords:** Motor Performance, Muscle Strength, Pelvis, Gait Speed, Stroke

## Abstract

**Background:** Poor motor recovery of hip muscles affect the walking post-stroke. The study objective was to examine how lower extremity motor function and hip muscle weakness are related to weight-bearing asymmetry (WBA), excessive pelvic tilt, and gait speed in stroke survivors.

**Methods:** Eighty patients with chronic stroke, a mean and standard deviation (SD) of post-stroke duration of 350 ± 664 days, age of 30-70 years, independent standing, and 10-meter walking capacity participated in the study. Hip muscular strength was measured using a handheld dynamometer (HHD) and motor function was assessed by Fugl-Meyer Assessment of lower extremity (FMA-LE). The WBA was recorded using two weighing scales; whereas the pelvic tilt and gait speed were evaluated using palpation meter (PALM) and 10-meter walk test, respectively.

**Results:** The muscles strength of hip flexors, extensors, abductors, and adductors of the paretic side ranged between 22.0 and 24.4 pounds. The mean score of FMA-LE was 22 points. Following Pearson product-moment correlation with statistically significant P < 0.05, the relationship of hip muscles strength and FMA with WBA, lateral pelvic tilt (LPT) and anterior pelvic tilt (APT), and speed are as follows: flexors (r = 0.47, r = 0.31, r = 0.44, r = 0.44), extensors (r = 0.45, r = 0.38, r = 0.37, r = 0.35), abductors (r = 0.49, r = 0.32, r = 0.38, r= 0.40), adductors (r = 0.45, r = 0.31, r = 0.23, r = 0.34), and motor function (r = 0.62, r = 0.33, r = 0.38, r = 0.62).

**Conclusion:** Motor performance of the paretic lower limb was highly correlated with WBA and gait speed in stroke survivors. Overall hip muscle strength of paretic side had a moderate correlation with WBA, excessive pelvic tilt, and gait speed.

## Introduction

Stroke is one of the largest causes of disability and half of all stroke survivors experience a major limitation in daily functioning.^1^ Clinical features of stroke include strength deficits, sensory-motor impairment, spasticity, incoordination, and postural dysfunction. Impaired motor control and weakness of extremities after stroke not only reduce the muscular force production but also affect the inter-limb coordination of functional movements.^2^ Muscle strength of the affected lower extremity is usually reduced by 34%-62% in patients post-stroke compared to healthy individuals.^3^

While standing, the majority of stroke survivors bear only 25%-43% of their body weight on the affected lower limb.^4^ In response to an external perturbation, an increased response or early recruitment of the unaffected adductor for compensating poor and delayed response of the affected muscles was observed in patients post-stroke.^5^ They further rely more on the unaffected leg to keep their balance, allowing for asymmetric weight-bearing between feet. Poor dissociated movement between trunk and pelvis or reduced lower limb motor functioning might account for asymmetric weight distribution between feet in ambulatory community stroke patients.^6^ Excessive pelvic tilt while sitting was related to poor trunk performance in stroke.^7^ In addition, an altered pelvic alignment in standing was related to weight-bearing asymmetry (WBA) and motor recovery of lower limbs.^8^ It was believed that the pelvic stability of stroke survivors is influenced by hip muscle weakness and the motor recovery of the paretic lower limb. Weakness of hip abductors and adductors were shown to be affecting the pelvic stability of weight-bearing lower extremity during functional mobility, accounting for the poor balance control in medial-lateral directions. Stroke survivors having difficulty in transferring the body weight towards paretic side due to pelvic instability usually generate insufficient force production of hip flexors and extensors to propel the body forward during gait.^9^^,^^10^

Lateral displacement of the pelvis during walking was large and oriented more over the sound side which might be attributed to poor motor control and weakness of hip muscles in stroke survivors.^11^ The vertical displacement of the most affected side of pelvis and an increased anterior pelvic tilt (APT) might further be related to poor lower trunk control and weakness of hip extensors and abductors. The weakness of hip muscles has been proven to be limiting the gait speed in ambulatory stroke patients.^12^^,^^13^ Inadequate weight-bearing capacity of the most affected lower limb was directly related to gait parameters.^14^ Despite the importance of motor function and hip muscles strength of paretic lower extremity on the weight-bearing capacity between feet and walking capacity in individuals post-stroke, the extent to which these variables affect the pelvic stability, WBA, and gait speed is not well understood, so there was a need to conduct this study.

## Materials and Methods

This cross-sectional study received approval from the Institutional Research Committee of Manipal College of Health Professions, Manipal Academy of Higher Education, Bangalore, India, and got registered in the Clinical Trials Registry of India (CTRI/2017/09/009166). Chronic stroke survivors of at least 4-month post-stroke duration were contacted and explained about the purpose, benefits, and brief methodology of the study. All the volunteers signed an informed consent form prior to their participation in the study. Following eligibility screening, they were recruited from tertiary care rehabilitation centers, community settings, and outpatient physiotherapy departments of specialty hospitals. They were undergoing physiotherapy rehabilitation strategies involving treatment of spasticity, strengthening exercise for paretic lower limb muscles, and balance and mobility training. Patients of both gender aged between 30 and 70 years with first-ever ischemic or hemorrhagic stroke, independent standing and walking ability of at least 10 meters distance, medical stability, no brainstem and/or cerebellar stroke, no visual field defects, and Brunnstrom lower limb recovery stage of ≥ 3 were included in the study. Patients were excluded from the study if presented with peripheral vestibular dysfunction, absent light touch and proprioception of the paretic foot, any perceptual dysfunction resulting difficulty in understanding verbal instructions, any diagnosed musculoskeletal dysfunction of lower extremities and injection of botulinum toxin in the spastic quadriceps and plantar flexors muscles in the recent 4-month period affecting their standing posture and walking, and knee hyperextension beyond 30 degrees resulting from the contracture of hip flexors and ankle plantar flexors. 

**Figure 1:**
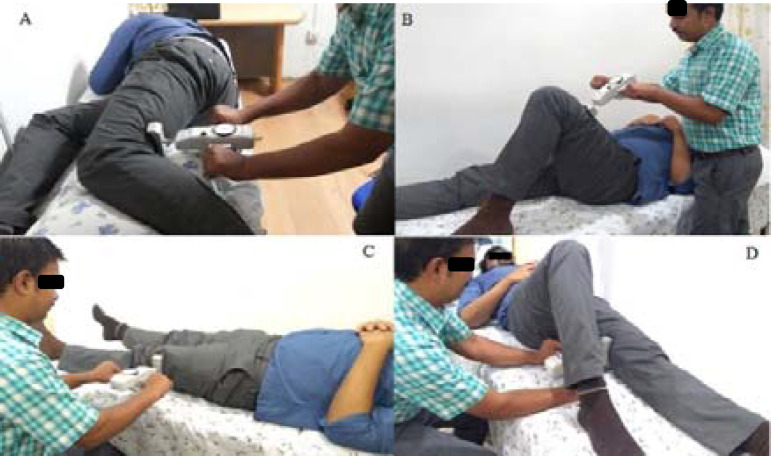
Hip muscle strength testing using handheld dynamometer (HHD); A. extensors, B. flexors, C. abductors, D. adductors

***Clinical measures:*** Fugl-Meyer Assessment of lower extremity (FMA-LE), a reliable clinical utility tool, was used to assess the motor function of the paretic side post-stroke.^15^ The FMA-LE with the maximum score of 34 points evaluates the reflex activities in supine and sitting positions, basic lower limb muscle synergies, movement combining synergies, out of synergies, and coordination/speed of the leg movement. Isometric muscle strength (lb) of hip flexors, extensors, abductors, and adductors was tested by break test using handheld dynamometer (HHD). HHD was proven to be reliable for measuring muscle strength in patients after stroke.^16^^,^^17^ The test positions and procedures were standardized.^18^^,^^19^ The patient’s hip extensors strength was measured in a side-lying position. The top leg was kept over a pillow and the bottom leg was flexed at hip and knee to 90°. The HHD was placed posterior to the testing leg at the distal thigh and the patient was then asked to extend the hip by generating maximum force against the dynamometer. The hip flexors strength was tested in supine position, the patient’s testing leg being flexed at knee with foot flat on the plinth. The patient was instructed to flex the hip by sliding the foot toward the pelvis while generating the maximum force against dynamometer kept at the anterior aspect of distal thigh. To test the hip abductors and adductors muscle strength in supine lying, the patient was instructed to generate maximum force against the dynamometer placed at lateral and medial aspect of the distal part of thigh, respectively (Figure 1).^19^

**Figure 2:**
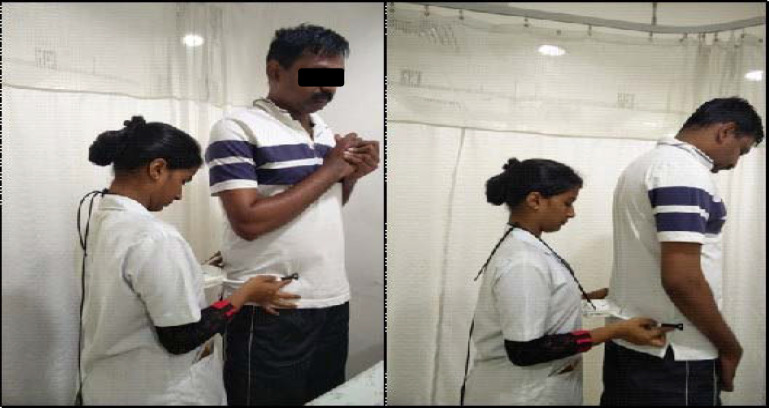
Measurement of anterior pelvic tilt (APT) and lateral pelvic tilt (LPT) using palpation meter (PALM) device

The excessive pelvic tilt in standing was tested using a palpation meter (PALM) device, a portal and reliable tool applicable in clinical settings. For recording the excessive anterior-posterior tilt whilst the patient was in erect standing posture, the anterior superior and posterior superior iliac spines were initially palpated and marked on the skin. The caliper tips of PALM instrument were positioned at the marked points and the degree of excessive APT was then recorded by the physiotherapist standing beside the patient. To record the lateral pelvic tilt (LPT), the therapist stood behind the patient and placed the caliper tips at the most superior part of the iliac crests. The nearest angle of LPT was read off in the inclinometer (Figure 2).^20^

The WBA between feet in patients post-stroke was tested using two standard weighing scales. Following the validation of two standard weighing machines using metal weights, the patient was asked to look straight and stand erect by placing the feet on the two weighing machines. The WBA was recorded by subtracting the total body weight upon weight on the most affected leg minus least affected leg. The inter-rater and intra-rater reliability was demonstrated to be excellent (r = 0.94, r = 0.97, respectively).^21^^,^^22^ The gait speed was measured using a 10-minute walk test. The patient was instructed to walk at self-selected speed over a 10-meter walkway and the middle six-meter distance was considered for analyzing the walking speed (distance traveled divided by time taken) to overcome the acceleration and deceleration effects.^23^ The cadence was recorded by counting the number of steps in a minute.

## Results

The demographic characteristics of the study participants are presented in table 1. The mean and standard deviation (SD) of age of the patients was 56.7 ± 12.6 years and the post-stroke duration was 350 ± 664 days.

**Table 1:** Demographic characteristics of patients with stroke (n = 80)

**Characteristics**	**Value**
Age (year) (mean ± SD)	56.7 ± 12.6
Gender [n (%)]	
Male	54 (68)
Female	26 (32)
Type of stroke [n (%)]	
Hemorrhagic	13 (16)
Ischaemic	67 (84)
Time since a stroke (day) (mean ± SD)	350 ± 664
Paretic side [n (%)]	
Left	28 (35)
Right	52 (65)
Brunnstrom’s lower limb recovery stage [n (%)]	
III	14 (16)
IV	55 (64)
V	11 (13)
MAS [n (%)]	
Quadriceps 1, 1+, 2	12 (15), 48 (60), 20 (25)
Plantar flexors 1, 1+, 2	19 (23), 34 (43), 27 (34)

MAS: Modified Ashworth scale; SD: Standard deviation

64% of them were men and 84% suffered from ischaemic stroke. 65% of the patients presented with right-sided paresis and 64% were in Brunnstrom’s lower extremity motor recovery stage 4. In the modified Ashworth scale (MAS), a score of 1+ point for the quadriceps was seen in 60% of patients and 43% for the plantar flexors muscles. Only 15% and 23% of study participants presented a score of one point in the quadriceps and plantar flexor muscles.

The hip muscle strength on the paretic side is given below: flexors 23.0 ± 5.7, extensors 22.4 ± 6.3, abductors 24.4 ± 5.5, and adductors 24.4 ± 6.3 pounds. Lower extremity motor function was 22.1 ± 5.4 points (ranged from 6 to 32 points) as measured by FMA-LE out of maximum possible score of 34 points. There was an increased APT and LPT, ranging up to 9 degrees with a mean and SD of 5.7 ± 1.5 and 3.8 ± 1.4 degrees, respectively. WBA was 0.22 ± 0.15 with a score of one indicating symmetrical weight-bearing between feet. The gait speed was 0.49 ± 0.24 m/s and the cadence was 73 ± 13 steps per minute. The motor function of the lower limb and hip muscle strength in stroke survivors and their relationship with pelvic obliquity, WBA, and gait speed are presented in table 2.

## Discussion

The objective of this study was to test the relationship of motor function of the paretic lower limb and hip muscle weakness with pelvic tilt, WBA, and gait speed in stroke survivors. The lower limb motor performance measured using FMA-LE had a high correlation with WBA and gait speed in patients post-stroke. All the hip muscular strengths of the paretic side had a moderate correlation with WBA.

**Table 2:** The motor function of lower limb and hip muscle strength in stroke survivors and their relationship with pelvic tilt, weight-bearing asymmetry (WBA), and gait speed (n = 80)*

**Clinical measures**	**LPT (degree)**	**APT (degree)**	**WBA**	**Cadence**	**Gait speed**
FMA-LE	-0.330	-0.387	-0.620	0.546	0.619
Hip strength (lb) flexors	-0.309	-0.439	-0.475	0.474	0.443
Extensors	-0.376	-0.389	-0.455	0.367	0.345
Abductors	-0.319	-0.382	-0.489	0.416	0.404
Adductors	-0.314	-0.235	-0.454	0.336	0.339

FMA-LE: Fugl-Meyer Assessment of lower extremity; WBA: Weight-bearing asymmetry; APT: Anterior pelvic tilt; LPT: Lateral pelvic tilt

The “r” was interpreted as very low (< 0.20), low (0.20-0.39), moderate (0.40-0.59), high (0.60-0.79), and very high (0.80–1.00), respectively;

* P-value < 0.05 was statistically significant

We also observed that there was a low moderate correlation of movement performance of lower extremity and hip muscles strength with APT and LPT. The strength of hip adductors and extensors was low moderately correlated with cadence and gait speed; whereas the flexors and abductors showed a moderate correlation with the same measures.

The mean score of 22 points out of a maximum of 34 points in the FMA-LE reflects the poor timing of motor activation and abnormal sequencing of paretic lower limb motor recovery in our study participants. Hsu et al.^24^ and Nadeau et al.^25^ observed similar findings in their work. Reduced recovery pattern of hip abductors and adductors in standing after stroke is majorly responsible for side to side balance stability. The paralyzed leg muscles showed inadequate and delayed motor response to anticipatory postural perturbations and they rely heavily on the contralateral muscles to maintain balance.^5^ The movement combining synergies of the lower limb observed in FMA-LE during the non-weight bearing and weight-bearing situations should demand selective muscle control at the proximal joints of lower limb. The weight-bearing capacity between feet is not only influenced by motor performance of the lower limb, but also by the strength of hip muscles. We observed that the hip muscle strength of the affected side was reduced by 50%-60%. This is in line with Dorsch et al.^3^ who reported 34%-62% reduction in the hip muscle strength on the affected lower limb in chronic stroke. 

In the current study, WBA was reduced by 22% compared to 21% reported by Adegoke et al.^26^ in chronic stroke. The high correlation of FMA-LE and moderate correlation of hip muscle strength to WBA in patients post-stroke might be related to proximal dynamic instability and poor selective motor performance of paretic lower limb in standing. Sackley^27^ found a similar correlation of FMA-LE with WBA in chronic stroke. The motor performance and strength of hip abductors and adductors are important for maintaining the neutral pelvic alignment in the frontal plane; whereas flexors and extensors should contribute to pelvic stability in the sagittal plane. De Quervain et al.^28^ observed that patients who scored less in the FMA-LE presented with restricted pelvic motions while walking. Furthermore, the paretic hip muscles are responsible for side to side balance stability in standing.^5^ With numerous hip muscles being attached to the pelvic bone and lumbar spine, the proximal dynamic stability of pelvis is further determined by the coordinated activity between the lower trunk and hip muscles.^4^ Study on pelvic obliquity in patients post-stroke showed a moderate correlation to WBA.^8^ The finding of low moderate correlation of FMA-LE and hip muscle strength to altered pelvic tilt in patients with stroke shall support our assumptions. 

Motor performance of lower limb within 72 hours following the acute stroke is the best predictor of gait performance.^29^ Non-ambulant patients who regained sitting balance recovery with better trunk performance and some voluntary movement of the paretic lower limb within the first 72 hours post-stroke had a predictive value of 98% chance of independent walking ability within 6 months. Those who had poor trunk control and complete muscle paralysis of the leg during inpatient acute stroke care could show only 27% of predictive capacity of independent walking at 6-month follow-up.^30^ We observed a mean gait velocity of 0.49 m/s which is closely related to the category of household walkers post-stroke.^31^ The high correlation of the lower limb motor function and hip muscle strength which we observed with gait speed could be related to stance instability and inadequate leg advancement of the paretic side and poor trunk control, thus affecting the overall walking speed. Our views are supported by Hsu et al.^24^ and Nadeau et al.^25^ who reported a positive correlation of hip flexor muscle strength with gait velocity (r = 0.83, r = 0.54, respectively) in patients with stroke. Additionally, we observed that the weakness of hip extensors and flexors had a moderate correlation with gait speed. Cruz et al.^12^ confirmed that there was a positive correlation between hip extensor strength and gait velocity. However, we did not measure the trunk control in the current study.

The study results should be carefully interpreted due to the reasons mentioned below. First, we measured only isometric strength of all the hip muscles, but the isotonic muscle strength was not known. Hip flexors and abductors being the phasic muscle groups, the correlation of isokinetic muscle strength to pelvic stability, and gait speed post-stroke might provide additional information in future studies. Second, the findings cannot be generalized as the majority of the study participants (64%) were in the Brunnstrom’s stage 4 for the lower limb motor recovery. Third, the severity of spasticity in the lower limb muscles was not correlated with the walking speed. Fourth, stratified sub-group anlaysis was not conducted based on use of mobility aid and pathological gait pattern. Finally, we did not measure the lower trunk performance post-stroke. Had we measured the lower trunk muscle performance, it would have given us more understanding about the pelvic stability and WBA between feet in standing. In a previous study, the trunk control post-stroke showed a moderate correlation to pelvic tilt in standing and the correlation was high during the recovery stage of 5 for the lower limb.^32^ We recommend that future pelvic stability interventional trials in patients post-stroke should focus on the strategies emphasizing the lower trunk control, motor performance of the paretic lower extremity, and hip muscle strength.

## Conclusion

Motor performance of the paretic lower limb was highly correlated with WBA and gait speed in stroke survivors. Overall hip muscle strength of paretic side had a moderate correlation with WBA, excessive pelvic tilt, and gait speed.

## Notes:

**How to cite this article:** Darak V, Karthikbabu S. Lower limb motor function and hip muscle weakness in stroke survivors and their relationship with pelvic tilt, weight-bearing asymmetry, and gait speed: A cross-sectional study. Curr J Neurol 2020; 19(1): 1-7.
